# *Artemisia baimaensis* allelopathy has a negative effect on the establishment of *Elymus nutans* artificial grassland in natural grassland

**DOI:** 10.1080/15592324.2022.2163349

**Published:** 2023-01-16

**Authors:** Hang Yang, Jianchao Song, Xiaojun Yu

**Affiliations:** a College of Grassland Science, Gansu Agricultural University, Lanzhou, China; b Key Laboratory of Grassland Ecosystem, Ministry of Education, Gansu Agricultural University, Lanzhou, China; c Pratacultural Engineering Laboratory of Gansu Province, Sino-U.S, Lanzhou, China

**Keywords:** *Elymus nutans*, *Artemisia baimaensis*, litter aqueous extracts, volatile organic compounds, allelopathy, seed germination, seedling growth, physiological characteristics, artificial grassland establishment

## Abstract

Planting *Elymus nutans* artificial grassland to replace degraded *Artemisia baimaensis* grassland on the Qinghai Tibetan plateau (QTP) can effectively alleviate local grass-livestock imbalance. However, it is unknown whether the allelopathy of natural grassland plant *A. baimaensis* on *E. nutans* affects grassland establishment. Accordingly, we examined the effects of varying concentrations of aqueous extracts of *A. baimaensis* litter on the seed germination and early seedling growth of *E. nutans*, and the effects of *A. baimaensis* volatile organic compounds (VOCs) on the growth parameters and physiological characteristics of *E. nutans*. The results indicate that the aqueous extract inhibited the force, percentage, and index of germination of *E. nutans* and affected early seedling growth, particularly at high concentrations. Further, the VOCs significantly reduced the aboveground and root biomass of *E. nutans* and increased malondialdehyde concentrations. Additionally, these VOCs altered the antioxidant enzyme activities and increased the superoxide dismutase, peroxidase, ascorbic acid peroxidase, soluble sugar, and proline content but significantly decreased glutathione reductase levels. Our results indicate that the allelopathy of *A. baimaensis* significantly inhibited the germination and seedling growth of *E. nutans* . Thus, the leaching of *A. baimaensis* may produce allelochemicals in the soil that inhibit the germination of *E. nutans* seeds. Moreover, the VOCs of *A. baimaensis* may disrupt the growth process, resulting in a decrease in biomass and a disruption of the physiological metabolism of seedlings under field conditions.

## Introduction

The Qinghai-Tibetan plateau (QTP), known as the roof of the world and the water tower of Asia, is one of the most important terrestrial ecosystems.^1^ The predominant vegetation type of the QTP is natural grassland, which serves multiple ecological functions, such as soil and water conservation, acts as an animal habitat, and provides a material basis for the development of animal husbandry in alpine meadow.^2,3^ However, the degradation of grassland from long-term overgrazing has diminished grassland productivity and created poisonous weed communities and bare ground, making it insufficient to meet the needs of local livestock. To alleviate this situation, artificial grasslands are usually planted to replace degraded natural grasslands,^4^ but the interaction between poisonous weed in degraded grassland and artificial grassland is still unclear. Allelopathy is a phenomenon in which plants release chemicals into the environment to inhibit or stimulate nearby plants.^5^ This has an impact on individual performance, community structure, and plant invasion.^6^ Other ecological functions of allelopathy include altering plant defense functions and influencing organ resource allocation.^7^ Allelochemicals are released into the environment through decomposition, volatilization, leaching, and root exudation.^8,9^ Among these, foliar leaching was the most studied allelochemical source.^6^ Wang et al. reported that water extract of *Artemisia frigida* seedlings significantly inhibited the seed germination and root growth of *Lactuca sativa*,^10^ indicating that allelopathy of *A. frigida* is one of the primary mechanisms for it becoming a dominant species in degraded grassland. The aqueous extract of *A. frigida* inhibited the germination and early seedling growth of *Leymus chinensis, Stipa krylovii*, and *Cleistogenes squarosa*.^11^ Mahmoud et al. discovered that the wheat crops grown in the shade of *Jatropha curcas* trees had lower yields, and then demonstrated that the allelopathy of *J. curcas* leaf extract inhibited wheat seed germination under laboratory conditions, of which high concentration had the most significant effect.^12^

Plants release secondary metabolites according to the needs of a specific environment in the process of evolution, in which volatilization plays an important role.^13^ Plant volatile organic compounds (VOCs) are released constitutively or in response to stimuli and can perform a variety of ecological functions,^14^ such as affecting the growth and development, defense, and life cycle of surrounding plants.^15–17^ Meanwhile, volatilization is one of the main pathways involved in allelochemical release, and the VOCs are the primary medium of allelopathy between plants. Environmental stress caused by allelopathy will affect the morphological and physiological characteristics of plants.^18^ Previous studies found that herbivores induce plants to release VOCs and put recipient plants into a defensive state to reduce herbivore seasonal damage.^19,20^ When exposed to VOCs from the barley cultivar Alva, the Kara cultivar allocates more biomass to its roots compared with plants exposed to clean air.^21^ Muller et al. observed that the VOCs of the annual grassland species *Salvia shrubs* had a negative effect on the growth of recipient plants.^22^ In addition, allelopathy induces a burst of reactive oxygen species (ROS) in the target plants, which results in oxidative stress,^23^ such as O_2_^·-^ and H_2_O_2_. Highly reactive ROS disrupt plant metabolism via oxidative damage of lipids, proteins, and deoxyribonucleic acid, ultimately leading to programmed cell death.^24,25^ The antioxidant enzymes are directly involved in detoxification.^26,27^ Plant VOCs can affect the physiological characteristics of recipient plants, e.g., VOCs from garlic reduced superoxide dismutase (SOD) activity and increased hydrogen peroxide (H_2_O_2_) content in cucumber seedlings.^28^ VOCs released by the leaves of *Acacia dealbata* increased the SOD and peroxidase (POD) levels in *Lolium multiflorum* and the malondialdehyde (MDA) levels in *Trifolium subterraneum*.^29^ Chen et al. found that the volatile allelochemical α-pinene significantly altered SOD, POD, and APX enzyme activities of *Elymus nutans*.^30^

*E. nutans*, a perennial cool-season forage, is the predominant species for artificial grass planting in the QTP region because of its excellent quality and strong adaptability.^31^ *Artemisia baimaensis*, a plant of the *Artemisia* genus in the Asteraceae family and one of the degradation indicator species of grassland. The plant is not eaten by livestock and has a large patchy distribution, which leads to the reduction of the productivity of edible grass in the grassland. In order to solve this problem, local herdsmen directly reseed *E. nutans* on degraded grassland of *A. baimaensis*, or use artificial grassland of *E. nutans* to completely replace degraded grassland of *A. baimaensis* after plowing in their own pastures. However, we found that after the establishment of *E. nutans*, its growth environment was filled with a distinct, pungent odor throughout its growth period. We found the odor as a VOCs produced by *A. baimaensis* in natural grassland. At the same time, this phenomenon gives us an enlightenment, whether the *E. nutans* artificial grassland is affected by the release of allelochemicals from *A. baimaensis* under the leaching and volatilization pathways. Few studies have been conducted to determine whether the allelopathy of poisonous weed, specifically the allelopathy of *A. baimaensis*’s litter aqueous extracts, and living volatiles, will affect the establishment of artificial grassland in natural grassland. Therefore, we hypothesized that the allelopathy of *A. baimaensis* would affect the establishment of *E. nutans* artificial grassland in natural grassland of degradation.

Under laboratory conditions, we examined the effects of varying concentrations of *A. baimaensis* litter extract on the seed germination and seedling growth of *E. nutans*, as well as the effects of living *A. baimaensis* plants on the seedling growth and physiological characteristics of *E. nutans*. This study aimed to determine whether the allelopathy of *A. baimaensis* in natural grassland affects the quality of *E. nutans* artificial grassland.

## Materials and methods

### Materials

*A. baimaensis* live plants and litter were collected from the natural grassland population in Dawu Township, Maqin County, Qinghai Province, China (100°26’–100°43ʹN, 34°17’–34°25ʹE; above sea level). In July 2021, live *A. baimaensis* plants of uniform size and in the jointing stage were excavated along with the soil adhering to their roots to ensure successful transplantation. *E. nutans* seeds were purchased from Nongfeng Seedling Technology Development Company (Lanzhou City, Gansu Province, China) in May 2021 and stored at 20°C in the College of Grassland Science, Gansu Agricultural University. The soil was excavated from the forage experimental base of Gansu Agricultural University and stirred repeatedly to ensure uniformity.

### Experimental design

To determine whether *A. baimaensis* affects *E. nutans* via allelopathy, we conducted two experiments using Petri dishes incubation and soil culture. For the litter aqueous extract bioassay, four concentration treatments (0, 0.01, 0.02, and 0.05 g mL^−1^) were established, and for the VOCs bioassay, two concentration treatments (with and without *A. baimaensis*) were established.

### Litter aqueous extract bioassay

The purpose of this experiment was to directly determine the allelopathic effects of an aqueous extract of *A. baimaensis* litter on the seed germination and seedling growth of *E. nutans*. The litter was brought to the laboratory, washed with distilled water, and air-dried before being cut into 1 cm fragments. The litter aqueous extracts were prepared following the methods of Mahmoud et al. and Wang et al.^12,32^ Briefly, 1, 2, and 5 g of litter were weighed and soaked in 100 mL of distilled water in the glass bottle, which was maintained at room temperature for 48 h (the concentration selection is based on the plant growth phenotype obtained from the previous short-term pre-experiment results). The extracts were then filtered through 0.45 mm filter paper to obtain 0.01, 0.02, and 0.05 g of *A. baimaensis* litter aqueous extract per milliliter. All extracts were stored at 4°C until and during use, and the extracts were prepared again every three days.

*E. nutans* seeds were sterilized with 0.01% sodium hypochlorite for 10s and rinsed six times with distilled water. Then, 50 seeds of *E. nutans* were selected and placed on the two layers of filter paper in a petri dish with a diameter of 12 cm. Five milliliters of each concentration of aqueous extracts of *A. baimaensis* litter were added, and 5 mL of distilled water served as the control. Then, five petri dishes of each treatment were placed in an incubator with a day/night temperature of 25°C/20°C and 14/10 h light/dark cycles (HGZ-HS250, Shanghai Hengyue Medical Instrument Co., Ltd., China). The number of germinated seeds was monitored daily, and the aqueous extracts were added. Radicle extension of 2 mm from the seed coat was defined as germination.

### Volatile organic compounds (VOCs) bioassay

**Figure 1.**
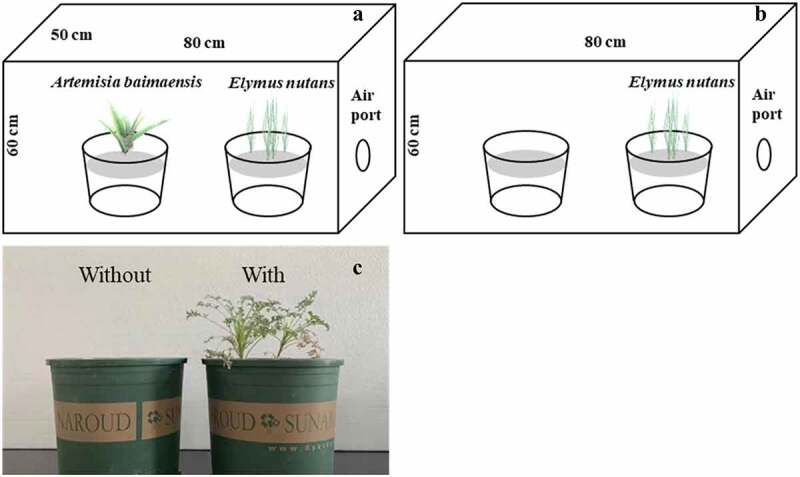
Growth of *E. nutans* with *A. baimaensis* (a) and without *A. baimaensis* (b), and transplanted *A. baimaensis* (c).

This experiment measured the impact of VOCs of *A. baimaensis* on the biomass and physiological characteristics of *E. nutans* during its growth. A single plant of *A. baimaensis* was transplanted into a pot with an upper diameter of 18 cm and a height of 19 cm, and it grew for 15 days. Five pots of plants with good growth and uniform size were selected for the experiment. Further, 50 sterile *E. nutans* seeds were chosen and planted in a 3 kg soil-filled pot. Then, the *A. baimaensis* pot and the *E. nutans* pot were regarded as a treatment and placed in the same semi closed transparent growth chamber of plastic, *E. nutans* pot and a pot with soil and no *A. baimaensis* served as the control (Figure 1a-c). There are 20 pots in two treatments (5 pots *A. baimaensis*, 5 pots soil, and 10 pots *E. nutans*), forming 10 growth groups (5 *A. baimaensis-E. nutans* groups and 5 soil-*E. nutans* groups). All tests for each treatment were conducted in quintuplicate. Next, the growth chambers of each growth group were separately put into the culture chambers under the same conditions (day/night cycle: 14/10 h, day/night temperature: 25°C/20°C, relative humidity: 60 ± 5%, and photon density: 9000 Lux; HGZ-HS250, Shanghai Hengyue Medical Instrument Co., Ltd., China). The seedlings’ growth was monitored every day, and the same amount of distilled water was added to each pot to ensure normal growth. Then, the seedlings of *E. nutans* in each treatment were taken from their pots after 45 days and used for the following index measurement.

### Index measurement

#### Litter aqueous extract bioassay

The seed germination force (GF) was recorded on the 3 days, and the seed germination percentage (GP) was measured on the 10 days. On the 10 days, 10 seedlings for each treatment were randomly selected to measured shoot length (SL) and root length (RL) with a ruler. The germination index (GI), indices of allelopathic effects (RI),^33,34^ and synthetic allelopathic effect index (SE) were calculated by using the following equations (1)-(3),

(1) $$Germination\;index(GI)=\sum (\frac{Gt}{Dt})$$

(2) RI=1−C/T(T≥C)orT/C−1(T<C)

(3) $$\;SE=\frac{\;R{\;I}_{\;GF}\;+R{\;I}_{\;GP}\;+R{\;I}_{\;GI}\;+R{\;I}_{\;SL}\;+R{\;I}_{\;RL}}{\;5}$$

where Gt is the number of seeds emerging on any given day, Dt is the time after setting the seeds for germination; T is the treatment value, and C is the corresponding value for the control. Positive values of RI indicate a stimulatory effect, and negative values indicate an inhibitory effect of the aqueous extract.

### VOCs bioassay

#### Determination of the plant height, aboveground biomass, belowground biomass, and root-shoot ratio

Ten plants of *E. nutans* from each treatment were randomly selected to measure plant height. The aboveground and root biomass samples of *E. nutans* were oven-dried at 72°C to achieve a constant weight to measure aboveground biomass and root biomass (mg per plant). The root-to-shoot ratio was calculated as the ratio of root dry weight to aboveground dry weight.^35^ Further, the remaining aboveground fresh leaves of *E. nutans* for each treatment were stored at −80°C to determine the physiological indices.

#### Determination of the activities of antioxidant enzymes

The SOD activity was determined by the nitrogen blue tetrazole method, using the SOD detection kit (G0101F). The POD activity was determined by the guaiacol method, using the POD detection kit (G0107F). The CAT activity was determined by the ultraviolet absorption method, using the CAT detection kit (G0107F). The ascorbate peroxidase (APX) activity was determined using the APX detection kit (G0203F). The glutathione reductase (GR) activity was determined by the enzyme circulation method, using the GR detection kit (G0209F). All the kits for determination were purchased from Suzhou Grace Biotechnology Co., Ltd., China (http://www.geruisi-bio.com).

#### Determination of the contents of soluble sugar, soluble protein, and free proline

The content of soluble sugar of *E. nutans* seedlings was determined using the soluble sugar detection kit (G0501F, Suzhou Grace Biotechnology Co., Ltd., China, http://www.geruisi-bio.com). The determination of soluble protein was carried out with 1 g fresh sample, which was grinded in 5 mL extraction buffer. The mixture was agitated for 30 min on ice before being centrifuged at 10000 g for 10 min at 4°C, with the supernatant saved. The level of the soluble protein was determined at 595 nm with Coomassie Brilliant Blue G-250, and bovine serum albumin was used to prepare the standard curve.^36^ Free proline was extracted according to Bates et al. with some modifications.^37^ one  gram fresh sample was homogenized in 5 mL 3% sulphosalicylic acid on ice and the homogenates were centrifuged at 6000 g for 20 min at 4°C. Two milliliters supernatant were mixed for reaction with 2 mL glacial acetic acid and 4 mL acid ninhydrin in the tube. After incubation at 100°C for 30 min, the reaction was terminated on ice. Next, 5 mL of toluene was added to the reaction mixture, which was then shaken well and left for 5 min to allow sufficient extraction for the separation of the organic and water phases. The upper toluene was saved in a new tube for detection at the absorbance of 520 nm with toluene as blank. Finally, the proline concentration was determined by the standard curve and calculated as fresh weight.

#### Determination of the contents of MDA, superoxide anion free radical (O_2_^·-^), H_2_O_2_, and hydroxyl radical scavenging ability

The contents of MDA, O_2_^·-^, H_2_O_2_, and hydroxyl radical scavenging ability of *E. nutans* seedlings were determined using the following determination kits: G0109F for MDA content; G0116F for O_2_^·-^ content; G0112F for H_2_O_2_ content; G0125F for hydroxyl radical scavenging ability. All the kits for determination were purchased from Suzhou Grace Biotechnology Co., Ltd., China (http://www.geruisi-bio.com).

### Statistical analyses

We used SPSS 24.0 (Chicago, Illinois) to analyze the one-way analysis of variance (ANOVA) of each index in the litter aqueous extract bioassay, with the extract concentration as the factor. The significance of differences was tested using Fisher’s protected least significant difference test (LSD) with a P-value ≤ 0.05 set as statistically significant. In the VOCs bioassay, growth and physiological indices of *E. nutans* were analyzed using one-way SPSS 24.0 analyses. The differences between the means were compared by the t-test.

## Results

### Effects of aqueous litter extracts of *A.* *baimaensis* on seed germination and seedling growth of *E.* *nutans*

**Figure 2.**
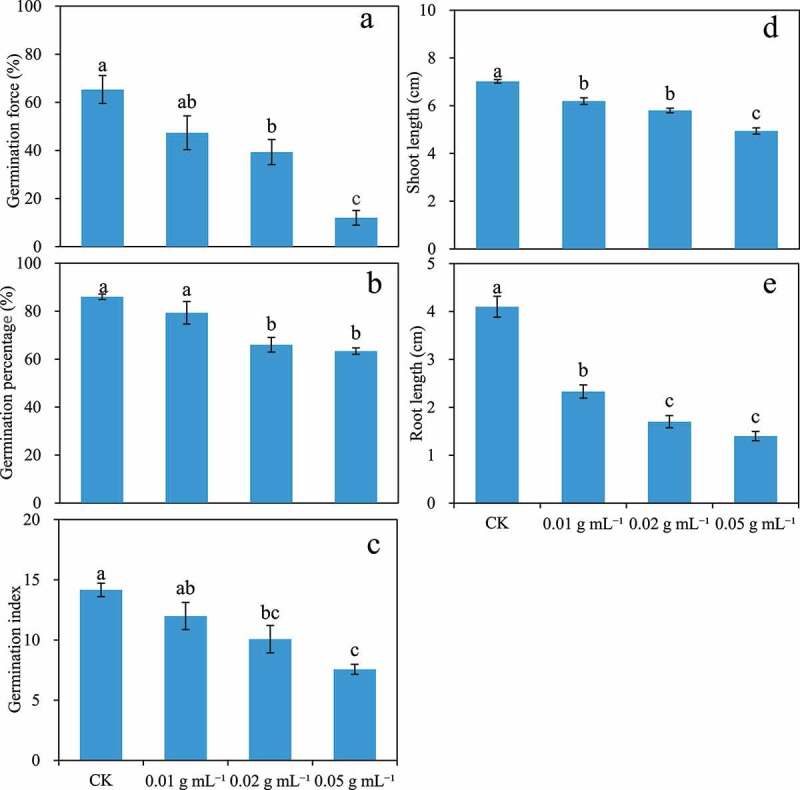
Effects of four different concentrations of aqueous litter extracts (CK, 0.01, 0.02, 0.05 g mL^−1^) collected from the *A. baimaensis* on germination force (a), germination percentage (b), germination index (c), shoot length (d), and root length (e) of *E. nutans*. Different letters indicate significant difference at different concentration treatment (P ≤ .05), and vertical bars indicate ± SE of mean.

The aqueous litter extracts of *A. baimaensis* led to decreased GF, GP, GI, SL, and RL of *E. nutans* (Figure 2a-e), which were negatively correlated with the concentration. The GF of *E. nutans* seeds under 0.02 and 0.05 g mL^−1^ treatments were significantly lower than that of the control by 39.8% and 81.63%, respectively. The GI of *E. nutans* seeds decreased by 28.9% and 46.58% compared with the control. With the increase of the concentration of aqueous litter extracts of *A. baimaensis*, the SL at concentrations of 0.01, 0.02, and 0.05 g mL^−1^ decreased significantly by 11.85%, 17.41%, and 29.65%, respectively, and the RL decreased significantly by 43.71%, 58.54%, and 65.85%.

**Figure 3.**
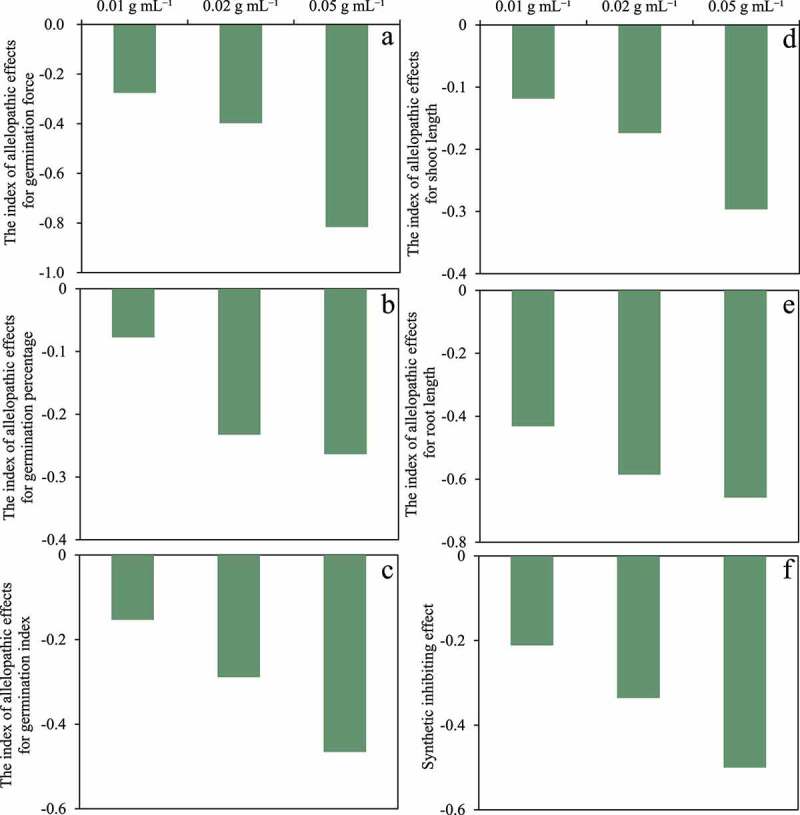
The effects of indices of allelopathic effects for the seed germination (A, B, C), seedling growth (d, e) indices, and synthetic inhibiting effect value (f) of *E. nutans* with four different concentrations of aqueous litter extracts (CK, 0.01, 0.02, 0.05 g mL^−1^) collected from the *A. baimaensis.*

The indices of allelopathic effects for GF, GP, GI, SL, and RL of *E. nutans* were less than zero under all concentration treatments (Figure 3a-e). The allelopathic effect indices decreased with the increase of concentration. Meanwhile, the synthetic allelopathic effect indices decrease with the increase of the concentration of litter extract and are all less than zero.

### Effects of VOCs of *A.* *baimaensis* on seedling growth of *E.* *nutans*

**Figure 4.**
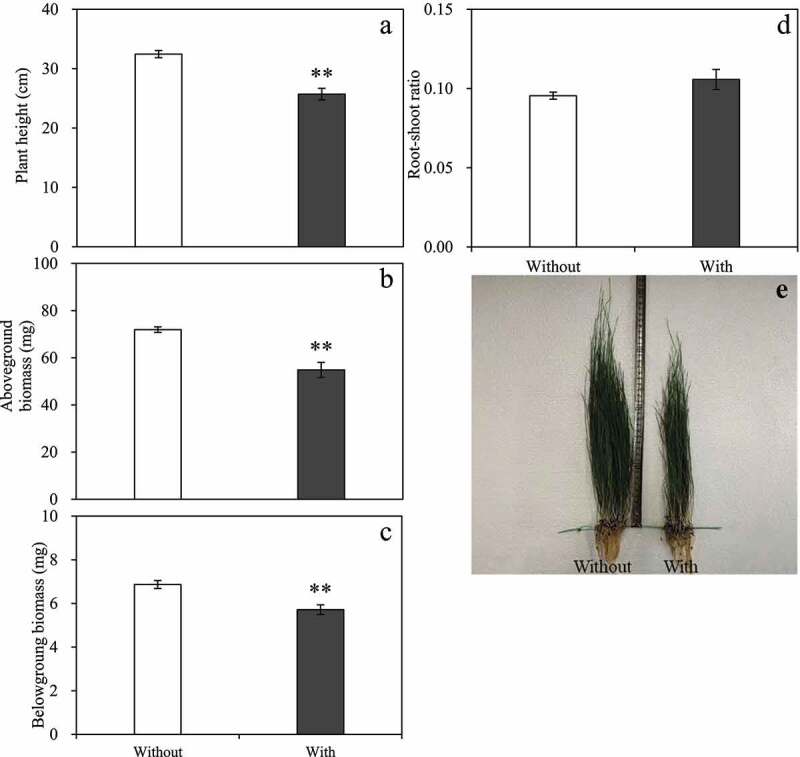
Effects of *A. baimaensis* VOCs on plant height (a), aboveground biomass (b), belowground biomass (c), root-shoot ratio (d), and phenotypes (e) of E. nutans. ** indicant significant difference than CK (P ≤ .01), * indicant significant difference than CK (P ≤ .05), and vertical bars indicate ± SE of mean.

*Baimaensis* VOCs significantly reduced the plant height, aboveground biomass, and root biomass of *E. nutans* but not the root-shoot ratio (Figure 4a-e). The plant height decreased by 20.75% (Figure 4a), the aboveground biomass decreased from 71.93 mg to 54.87 mg (Figure 4b), and the underground biomass decreased by 16.75% (Figure 4c). Under the treatment of *A. baimaensis* VOCs, the root-shoot ratio of *E. nutans* increased by 10.70% compared with the control (Figure 4d).

### Effects of VOCs of *A.* *baimaensis* on levels of lipid peroxidation and of *E.* *nutans*

**Figure 5.**
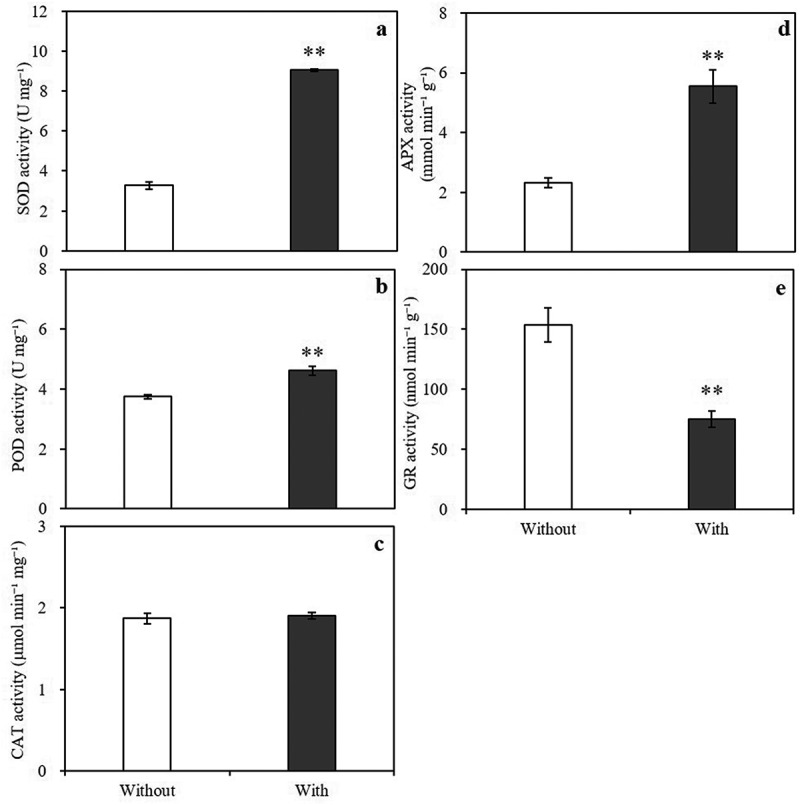
Effects of *A. baimaensis* VOCs on activities of SOD (a), POD (b), CAT (c), APX (d), and GR (e) of E. nutans. ** indicant significant difference than CK (P ≤ .01), * indicant significant difference than CK (P ≤ .05), and vertical bars indicate ± SE of mean.

**Figure 6.**
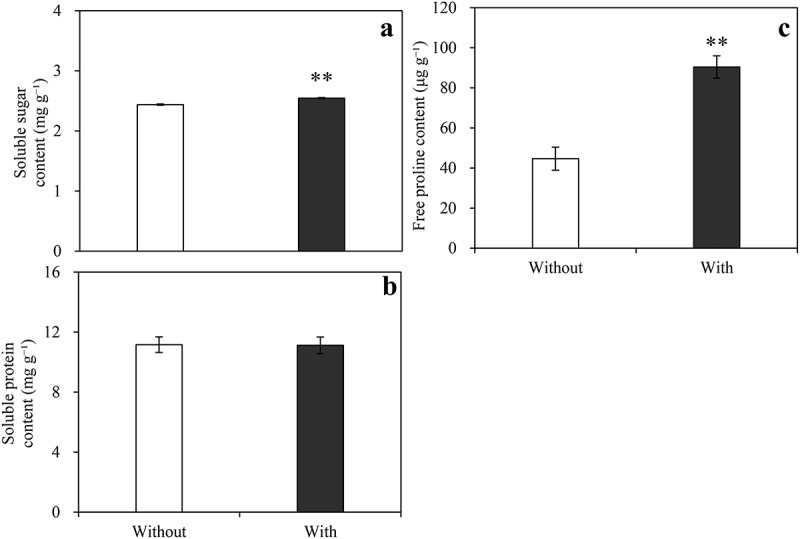
Effects of *A. baimaensis* VOCs on contents of soluble sugar (a), soluble protein (b), and free proline (c) of E. nutans. ** indicant significant difference than CK (P ≤ .01), * indicant significant difference than CK (P ≤ .05), and vertical bars indicate ± SE of mean.

Compared with the control, *A. baimaensis* VOCs significantly increased the SOD, POD, and APX enzyme activities of *E. nutans* but not the CAT enzyme activity. Among them, the SOD, POD, and APX enzyme activities of *E. nutans* increased by 176.76%, 23.13%, and 139.17% compared with the control, respectively (Figure 5a, b,d). The GR enzyme activity decreased by 51.27% (Figure 5e). *A. baimaensis* VOCs affected the content of osmotic regulatory substances in *E. nutans* (Figure 6a-c). The contents of soluble sugar and proline in *E. nutans* were significantly increased by 4.44% and 102.47% compared with the control, respectively.

### Effects of VOCs of *A.* *baimaensis* on levels of lipid peroxidation and hydroxyl radical scavenging ability of *E.* *nutans*

**Figure 7.**
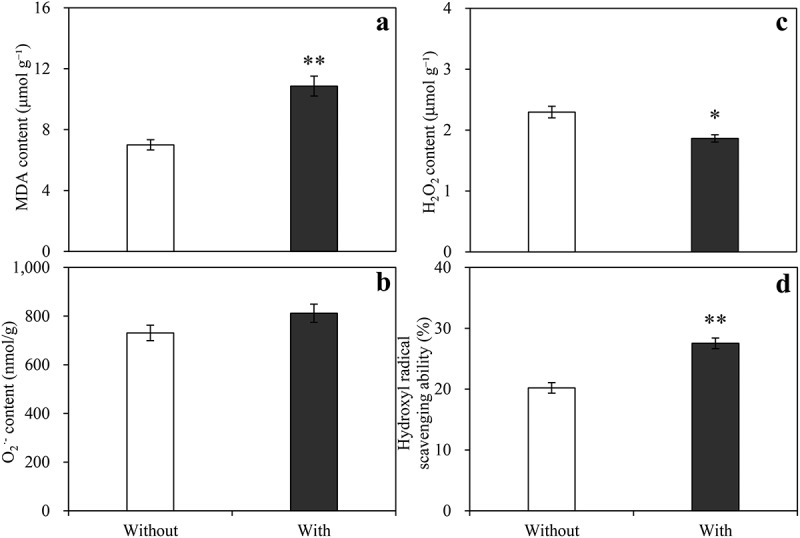
Effects of *A. baimaensis* VOCs on contents of MDA (a), O_2_^·-^ (b), H_2_O_2_ (c), and hydroxyl radical scavenging ability (d) of E. nutans. ** indicant significant difference than CK (P ≤ .01), * indicant significant difference than CK (P ≤ .05), and vertical bars indicate ± SE of mean.

*Baimaensis* VOCs significantly increased the MDA content and hydroxyl radical scavenging capacity of *E. nutans*, but there was no O_2_^·-^ (Figure 7a, b, d). H_2_O_2_ content was significantly reduced by 18.85% compared with the control, and MDA and hydroxyl radical scavenging capacity were increased by 55.17% and 36.27% compared with the control, respectively.

## Discussion

The allelochemicals released by donor plants can inhibit seed germination, seedling growth, or both in surrounding plants.^38,39^ The germination potential indicates the uniformity and vitality of plant germination. The seed germination rate indicates the amount of seed germination, and the germination index reflects the germination capacity and vitality of the seeds.^40^ Seed germination plays an important role in the reproduction and survival of plant populations. In the present study, the litter aqueous extract of *A. baimaensis* inhibited the seed germination and seedling growth (both the shoots and roots) of *E. nutans*, and the inhibitory effect increased with the increase of the concentration of the aqueous extract of the litter. These results were in agreement with those of previous studies that found a concentration gradient effect between seedling growth and active substances.^41–43^ In this study, the GF of *E. nutans* seeds treated with 0.05 g mL^−1^ aqueous extract of *A. baimaensis* litter was reduced by 81.63% compared to the control, while the GP of *E. nutans* seeds reduced by 26.36%. The results suggest that the seeds’ germination was delayed by the high concentration of the extract. In addition, the aqueous extract of the *A. baimaensis* litter had a greater inhibitory effect on the root growth of *E. nutans* than on their shoots. These results were supported by previous studies have found that a concentration gradient of aqueous extract of plant organs had an important effect on plant growth and seed germination,^12,42^ which also indicates that secondary metabolites in the litters of donor plants were leached and that allelochemicals affected the growth and development of recipient plants. This effect may be caused by the synergy, addition, or antagonism of multiple allelochemicals.^44,45^ The results of our litter aqueous extract bioassay indicated that the allelopathic effect of *A. baimaensis* may affect the seed germination and biomass of *E. nutans* in the field, and it is also necessary to further explore the allelopathy of *A. baimaensis* on *E. nutans* in combination with laboratory and field studies.

Plant VOCs are typical examples of secondary metabolites. Due to the physical characteristics of low molecular weight, these compounds are able to freely cross the cell membrane and be released into the environment.^46^ VOCs can be used as air signals to send signals over long distances to plants, allowing them to enter a defensive state.^47^ The VOCs emitted by plants can induce allelopathy and inhibit the growth of surrounding competing species.^48^ In the present study, the VOCs released by *A. baimaensis* inhibited the seedling growth of *E. nutans*. Plants under stress can improve their nutrient use efficiency by reducing aboveground biomass and increasing root biomass, and produce a higher root-to-shoot ratio to resist stress.^35,49^ Although the VOCs of *A. baimaensis* decreased the plant height, aboveground biomass, and root biomass of *E. nutans*, the inhibitory effect on the root was less than that of the aboveground part of the seedlings, resulting in an increase in the root-to-shoot ratio of *E. nutans*. These findings suggest that *E. nutans* has altered its organ allocation strategy in order to resist the allelopathy caused by the VOCs of *A. baimaensis.*

Antioxidant enzymes such as SOD, POD, CAT, APX, and GR are directly involved in detoxification.^50^ These enzymes play a crucial role in the removal of ROS. In the current study, the VOCs of *A. baimaensis* significantly increased the activities of SOD, POD, and APX in *E. nutans*, while decreasing the concentrations of O_2_^·-^and H_2_O_2_. In addition, the increased ability to scavenge hydroxyl radicals may have contributed to the decrease in H_2_O_2_ concentration. Hydroxyl radicals are considered to be the most prevalent ROS, and the ability to scavenge hydroxyl radicals is important for the protection of biological systems.^51^ Our results demonstrated that the allelopathy of VOCs from *A. baimaensis* causes *E. nutans* seedlings to increase the activities of a series of antioxidant enzymes that scavenge ROS.

The accumulation of MDA is a consequence of lipid peroxidation,^52^ and the MDA concentration is typically used to quantify the degree of membrane lipid peroxidation.^53,54^ *A. baimaensis’* VOCs increased the MDA concentration of *E. nutans*. The results indicated that the membrane lipid of the seedlings was damaged by ROS on membrane lipid, resulting in MDA accumulation. Even though the H_2_O_2_ concentration decreased in the present study, the seedlings still experienced stress from the ROS, which may be related to the significant decrease in GR enzyme activity in *E. nutans*. Gill et al. reported that GR enzyme activity improved the tolerance and antioxidant capacity of plants under abiotic stress.^55^

Osmoregulation is the maintenance of expansion pressure against stress by reducing the cellular osmotic potential, and the osmoregulatory substances are organic solutes synthesized by the cells themselves, such as soluble sugars, soluble proteins, and proline.^56^ In the present study, it was found that VOCs from *A. baimaensis* significantly increased the soluble sugar and proline contents of *E. nutans*, indicating that *E. nutans* has carried out osmotic regulation in response to the allelopathy of VOCs from *A. baimaensis*, thereby increasing its tolerance of *E. nutans*. Zhang et al. reported that the contents of proline and MDA in the recipient plant were increased by *Ageratina adenophora* VOCs.^57^ The results of our experiment demonstrated that the VOCs of *A. baimaensis* decreased the biomass of *E. nutans*, which may be due to the changes in physiological characteristics of *E. nutans* caused by VOCs, such as the increase in soluble sugar, MDA, and free proline concentrations, which were significantly negative correlated with plant phenotype.^43^

The allelopathy of an aqueous extract of *A. baimaensis* litter and living plant VOCs had negative effects on the seed germination and seedling growth of *E. nutans* under the laboratory condition. Therefore, it is important to consider the allelopathy of *A. baimaensis* when cultivating *E. nutans* artificial grassland in alpine meadows containing *A. baimaensis*. Although our experiments have demonstrated that the allelopathy of *A. baimaensis* inhibits the growth of *E. nutans*, there are still some limitations, such as the possible that *A. baimaensis* VOCs enter the environment via leaching, which may feed back to the recipient plants in natural grassland. Additional research involving the chemical recognition of allelochemicals from *A. baimaensis* and the effects of *A. baimaensis* litter and VOCs on the growth of *E. nutans*, soil biological community, and physical and chemical characteristics in the field is required.

## Conclusion

The results of this study demonstrated that the litter aqueous extract of *A. baimaensis* inhibited the seed germination and early seedling growth of *E. nutans*, and the VOCs released by *A. baimaensis* decreased the aboveground biomass and root biomass of *E. nutans*, while increasing the level of membrane lipid oxidation in *E. nutans* seedlings. These results strengthen the hypothesis that the allelopathy of *A. baimaensis* in natural grassland would affect the growth of *E. nutans* of artificial grassland.
